# Time trends in the prescription of statins for the primary prevention of cardiovascular disease in the United Kingdom: a cohort study using The Health Improvement Network primary care data

**DOI:** 10.2147/CLEP.S104258

**Published:** 2016-05-27

**Authors:** Aidan G O’Keeffe, Irwin Nazareth, Irene Petersen

**Affiliations:** 1 Department of Statistical Science, University College London, London, UK; 2 Department of Primary Care and Population Health, University College London, London, UK

**Keywords:** cardiovascular disease, primary prevention, statin therapy, time trend, United Kingdom

## Abstract

**Background:**

Statins are widely prescribed for the primary prevention of cardiovascular disease. Guidelines exist for statin prescriptions, but there is little recent analysis concerning prescription trends over time and how these vary with respect to demographic variables.

**Methods and results:**

Using The Health Improvement Network primary care database, statin therapy initiation and statin prescription prevalence rates were calculated using data from 7,027,711 individuals across the UK for the years 1995 to 2013, overall and stratified by sex, age group, and socioeconomic deprivation level (Townsend score). Statin therapy initiation rates rose sharply from 1995 (0.51 per 1,000 person-years) up to 2006 (19.83 per 1,000 person-years) and thereafter declined (10.76 per 1,000 person-years in 2013). Males had higher initiation rates than females and individuals aged 60–85 years had higher initiation rates than younger or more elderly age groups. Initiation rates were slightly higher as social deprivation level increased, after accounting for age and sex. Prescription prevalence increased sharply from 1995 (2.36 per 1,000 person-years) to 2013 (128.03 per 1,000 person-years) with males generally having a higher prevalence rate, over time, than females. Prevalence rates over time were generally higher for older age groups but were similar with respect to social deprivation level.

**Conclusion:**

The uptake of statins within UK primary care has increased greatly over time with statins being more commonly prescribed to older patients in general and, in recent years, males appear to have been prescribed statins at higher rates than females. After accounting for age and sex, the statin therapy initiation rate increases with the level of social deprivation.

## Introduction

Statins – a large class of cholesterol-lowering drugs – are widely prescribed for the primary prevention of cardiovascular disease (CVD).^1^,^2^ Since the 1990s, a number of clinical trials and large-scale studies have demonstrated the effectiveness of statins for CVD prevention^3^–^6^ and, in recent years, statins have become one of the most regularly prescribed medicines in the UK.^7^ In January 2006, the UK government’s National Institute for Health and Care Excellence (NICE) issued guidelines suggesting that statins should be prescribed by general practitioners (GPs – UK family doctors) to adults aged under 75 years whose risk of developing CVD within 10 years exceeds 20%.^8^ Generally, the 10-year risk of developing CVD is calculated using an established risk prediction method, such as the Framingham risk score^9^ or QRISK/QRISK2 risk calculators.^10^–^12^ In July 2014, this guideline was adjusted, with the statin prescription risk score threshold reduced to a risk of 10%. For the over 75s, the same NICE guidelines have recommended that statin prescriptions should be considered irrespective of the 10-year CVD risk score.

Clearly, statins have been commonly prescribed in the UK and the NICE guidelines have recommended their use for a wide section of the UK population. However, despite this, few studies have investigated the rate at which statin therapy is initiated, the prevalence of statin prescriptions, and how these may have changed over time. Some studies on trends in statins prescription in the UK were undertaken during the late 1990s and early 2000s, mainly using small data sources.^13^–^16^ Other past works have examined statin prescription trends within Europe,^17^–^24^ Canada,^25^ and the US,^26^–^28^ though, again, with either relatively small cohorts or not with the aim of examining statin prescription trends over time.

In this study, we attempt a comprehensive, large-scale assessment of prescribing trends for statins over time within the UK. We examine both the rate of statin therapy initiation (the rate at which subjects begin the use of statins) and the prevalence of statin prescriptions over time for the primary prevention of CVD. In addition, we examine possible differences in trends over time with respect to sex, age group, and level of socioeconomic deprivation. We use data from a large cohort of individuals that are generally representative of the UK population and have been collected over a long time period from 1995 to 2013.

## Data and methodology

### Data source

We used data from The Health Improvement Network (THIN) (www.epic-uk.org), a large-scale source of primary care data that is generally considered to be representative of the UK population.^29^,^30^ Approval for the use of THIN data for this research was granted by the Cegedim Strategic Data Medical Research UK Scientific Review Committee in August 2014 (approval number: 14–021). Because this study was based on data extracted from THIN, it was exempt from human subjects review, and members of the study population did not have to provide written informed consent. THIN data contain information on patient demographics alongside consultation information and diagnoses (indexed using Read codes)^31^ and patient prescription records. Data in THIN are linked longitudinally for each patient, which facilitates analyses of trends over time. Furthermore, THIN data include information on Townsend score, a measure of social deprivation using unemployment level, car ownership, home ownership, and household overcrowding levels to estimate the possible level of social deprivation within a particular postcode. Patients’ Townsend scores are categorized into five quintiles from 1 (least socioeconomic deprivation) to 5 (most socioeconomic deprivation).

### Study participants

We used data from general practices after the date at which they had adopted adequate methods for mortality reporting (acceptable mortality rate)^32^ and had a sufficiently developed computer system in use for patient data to be included in THIN (acceptable computer usage date).^33^ The study is on the use of statins for the primary prevention of CVD and, as such, only patients who had not previously experienced a CVD event were included in the cohort.

For analyses on the initiation of statin therapy, patients were included in the cohort from the earliest of: January 1, 1995, their 18th birthday, or registration with the practice until the earliest of: death, first CVD event, transfer from GP practice, first statin prescription, or December 31, 2013. Patients who initiated statins within 6 months of practice registration were excluded from analyses concerning initiation of statin therapy. This was done to ensure that patients who had moved GP practice but who had previously received statins would not be included. The use of such a 6-month “qualification period” has been routine in other studies concerning prescription trends using THIN data.^34^–^36^ For analyses on the prevalence of statin prescriptions, a 6-month qualification period was not used and patients were followed from cohort entry and beyond their first statin prescription.

### Statistical analysis

#### Initiation of statin therapy

We estimated the statin therapy initiation rate, that is, the rate of first statin prescription, per 1,000 person-years for each calendar year, over a time period from 1995 to 2013. Statin therapy initiation rates were estimated using data from all practices combined and also stratified by sex, age group, and Townsend deprivation score. Poisson regression models were used to estimate statin initiation rates, with log (person-years at risk) as an offset term, for each calendar year (1995–2013). We performed separate univariate analyses with each of: sex, age group, and Townsend score as explanatory variables and an overall multivariable analysis involving all of these variables. Age groups (in years) were classified as 18–34, 35–49, 50–59, 60–69, 70–74, 75–84, and ≥85.

#### Prevalence of statin prescriptions

We estimated the prevalence of statin prescription by considering “episodes” during which a given patient was receiving regular statin prescriptions. For each patient, a statin prescription episode occurs for a time period where statins were prescribed at least every 273 days (approximately equivalent to 9 months). A maximum time between successive prescriptions of 9 months was used since statins tend not to be prescribed for >6 months before a repeat prescription is issued. For each patient, in each calendar year of the cohort, the time periods during which a statin prescription episode occurs were calculated and summed over all to give an overall time during which patients were receiving statins for each year. We call this the “statin prescription time”. The time “at risk” (ie, the overall time for which patients were eligible to receive statins in each calendar year) was calculated and the statin prescription time was divided by the at risk time for each calendar year (and stratified by sex, age group, and Townsend score quintile, where appropriate) to calculate overall estimates of statin prescription prevalence. Statin prescription prevalence rates were rescaled and reported per 1,000 person-years.

Data manipulation and extraction were performed using Stata (version 14), with data analysis undertaken in R (version 3.1.2).

## Results

### Statin therapy initiation

**Figure 1:**
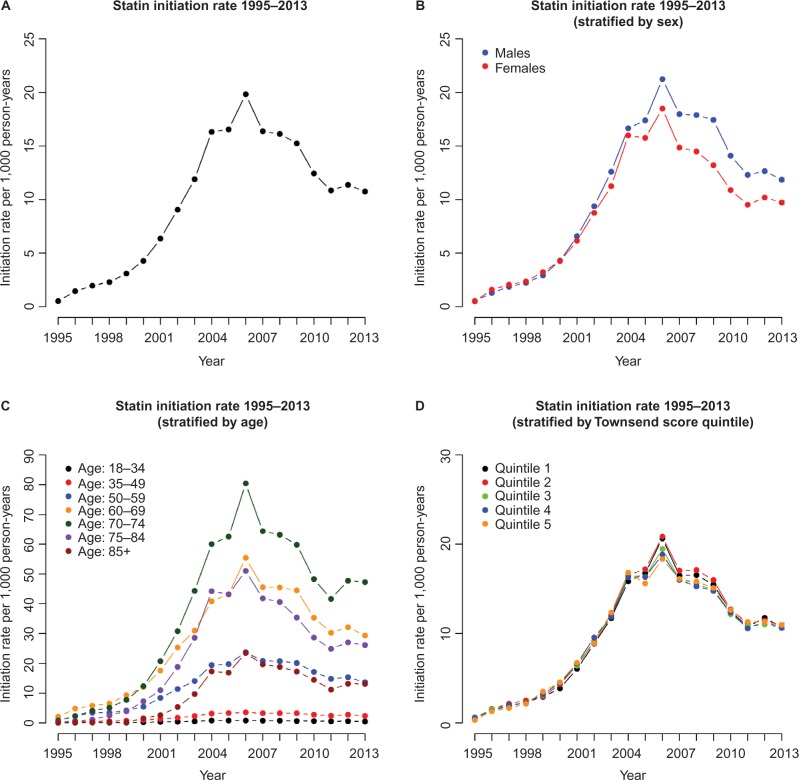
Plots showing the estimated statin therapy initiation rates from 1995 to 2013. **Note:** (**A**) Overall, (**B**) stratified by sex, (**C**) stratified by age group, and (**D**) stratified by Townsend score quintile.

Overall, there were 6,732,469 patients in our study of statin therapy initiation analyses of whom slightly more were female than male (52.3% and 47.7% of the cohort, respectively). The number of patients in each of the Townsend score quintiles 1 to 4 was broadly similar with fewer patients in the most deprived, fifth, Townsend score quintile (Table 1). This distribution is in line with that of Blak et al.^30^ Overall, statin therapy initiation rates rose at an increasing rate from 1995 (initiation rate =0.51 per 1,000 person-years) to 2004 (initiation rate =16.32 per 1,000 person-years), with a sharp peak at 2006 (initiation rate =19.83 per 1,000 person years), followed by a steady decline up to 2011 (initiation rate =10.88 per 1,000 person years) and, since 2011, rates have remained fairly constant (Figure 1A; Table 2). Where initiation rates are stratified by sex (Figure 1B; Table 2), a similar overall pattern is seen, although notably, since 2004, there appears to be a separation between males and females in that females generally have a slower rate of statin therapy initiation than males.

When statin therapy initiation rates were stratified by age group (Figure 1C), the rates remain low over time for those aged under 50 years, compared to other age groups, with the maximum statin therapy initiation rates (per 1,000 person years) of 0.83 for the 18–34 years age group (in 2005) and 7.88 for the 35–49 years age group (in 2006). For the 50–59 years age group, the rates are higher, in general, over time, approximately following a similar pattern to that seen in Figure 1A with an increase from 1995 (initiation rate =0.95 per 1,000 person-years) to 2006 (initiation rate =30.12 per 1,000 person-years) and a general decrease from 2007 to 2011 (initiation rate =17.95 per 1,000 person-years), with rates remaining steady since 2011. The 70–74 years age group has the highest statin therapy initiation rate for each year overall, with the maximum statin therapy initiation rate occurring in 2006 (initiation rate =80.42 per 1,000 person years). The 60–69 years and 75–84 years age groups also have high statin therapy initiation rates in each year, with maximum initiation rates (per 1,000 person years) of 61.90 and 51.01, respectively, in 2006. In contrast, statin therapy initiation rates were considerably lower for those in the 85 years and over age group, in each calendar year, with a maximum initiation rate of 23.45 per 1,000 person years in 2006.

Stratified by Townsend score quintile (Figure 1D), the initiation rates follow the same general pattern over time as the overall rates (Figure 1A), though there do not appear to be any obvious differences in initiation rates between the different Townsend score quintiles.

After adjusting for other demographic variables (multivariable model in Table 3), females were ~24% less likely to initiate statins than males (initiation rate ratio [IRR] and corresponding 95% confidence interval 0.76 [0.762 to 0.772]).

For age groups, relative to a fixed rate of 1 in the 50–59 years group, some age groups show comparably higher estimates (60–69 years: IRR 1.99 [1.973 to 2.002], 70–74 years: IRR 2.48 [2.546 to 2.504], and 75–84 years: IRR 1.57 [1.552 to 1.585]). Other age groups show comparably lower estimates (18–34 years IRR: 0.029 [0.028 to 0.029], 35–49 years: IRR 0.283 [0.281 to 0.286], and ≥85 years: IRR 0.69 [0.675 to 0.705]). For the Townsend score quintiles, IRR estimates, relative to the reference group of Townsend score quintile 1, were similar for quintiles 2 to 5, with estimates ranging from 0.98 (quintiles 3 and 4) to 1.03 (quintile 2) (univariable model in Table 3). However, after accounting for age group and sex (multivariable model in Table 3), the IRR estimates in the multivariable model increase with Townsend score quintile, with IRR estimates of: 1.05 (1.039 to 1.056), 1.14 (1.131 to 1.150), 1.26 (1.251 to 1.273), and 1.39 (1.372 to 1.399) for Townsend score quintiles 2, 3, 4, and 5, respectively.

### Prevalence of statin prescriptions

We consider results for the estimation of the prevalence of statin prescriptions. Table 4 shows a summary of the patients considered in the analysis of statin prevalence. There were slightly more females than males in the cohort (52.2% females and 47.8% males) with the distribution of Townsend score quintiles broadly similar to that for the cohort in Table 1.

**Figure 2:**
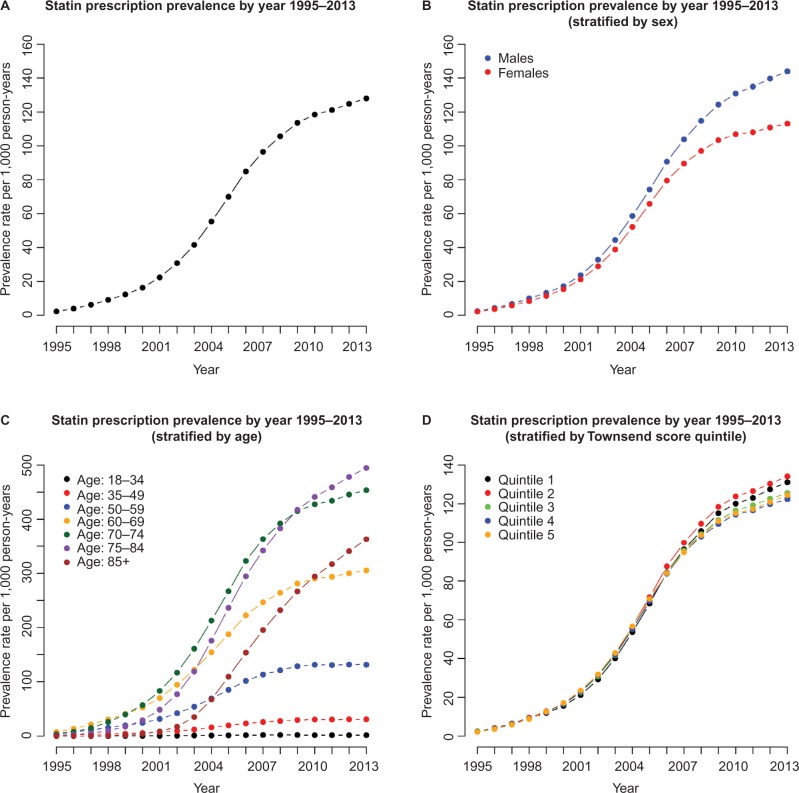
Plots showing the estimated statin prescription prevalence rates from 1995 to 2013. **Note:** (**A**) Overall, (**B**) stratified by sex, (**C**) stratified by age group, and (**D**) stratified by Townsend score quintile.

Overall, statin prescription prevalence was low in 1995 (prescription rate =2.36 per 1,000 person years) but rose slowly up to 2001 (prescription rate =22.41 per 1,000 person years), after which there was an exponential increase in prevalence up to 2007 (96.53 per 1,000 person years). Since 2007, although the statin prescription rate rises, the rate of increase has slowed somewhat (Figure 2A; Table 5). This same pattern is repeated for males and females separately (Figure 2B; Table 5), but we note that, since 2001, the rate of increase in prevalence in females has generally lagged behind that for males and the difference in prescription prevalence between males and females appears to be on the increase. This shift between males and females is similar to that seen for the statin therapy initiation rate in Figure 1B.

When statin prescription prevalence is stratified by age group (Figure 2C), prescription prevalence remains very low for the 18–34 age group, with a maximum prescription prevalence of 1.88 per 1,000 person years in 2008. For the 35–49 age group, prescription prevalence has tended to remain low, although the rate has risen in more recent years, increasing from 7.25 per 1,000 person-years in 2001 to 30.90 per 1,000 person-years in 2013. The change in the statin prescription rate has been more substantial for the 50–59 years age group, for whom prescription prevalence has increased from 4.96 per 1,000 person-years in 1995 to 131.56 per 1,000 person-years in 2013.

The oldest age groups (60–69, 70–74, 75–84, 85 and over) have experienced the greatest increase in statin prescription rate from 1995 to 2013. A sharp increase in the prescription rate was observed for each age group between 2000 and 2008. Since 2008, the increase in prescription prevalence has slowed slightly for the younger 60–69 years and 70–74 years age groups, with changes from 2008 to 2013 (per 1,000 person-years) of 264.00 to 305.18 and 392.15 to 453.77, respectively. In contrast, the prescription prevalence rate has continued to increase sharply for the 75–84 years and 85 years and over age groups with changes between 2008 and 2013 of 383.15 to 494.60 for the 75–84 years group and 232.03 to 390.70 for the 85 years and over group. At present, the 70–74 years age group has the highest statin prescription rate.

Up to 2007, there was little difference in satin prescription prevalence between the five Townsend score quintiles (Figure 2D). In 1995, rates varied from 2.15 per 1,000 person years for quintile 5 to 2.48 per 1,000 person-years for quintile 3. By 2007, rates for all Townsend score quintiles had increased substantially although little difference existed between quintiles, with 2007 rates ranging from 94.84 per 1,000 person-years in quintile 4 to 99.81 per 1,000 person-years in quintile 2.

In recent years, there appears to be a slight difference in prescription prevalence rates, with rates slightly higher in more affluent quintiles (quintiles 1 and 2) compared to less affluent quintiles (quintiles 4 and 5). In 2013, rates for quintiles 1 to 5 (per 1,000 person-years) were 131.12, 134.13, 125.56, 122.40, and 124.39, respectively. It is of note that prescription prevalence rates have increased gradually over time for each quintile, with no obvious sharp changes in prescription prevalence rates.

## Discussion

### Findings

The initiation rate of statin therapy increased sharply from 0.51 per 1,000 person-years in 1995 to 19.83 per 1,000 person-years in 2006, before declining slowly to a rate of 10.88 per 1,000 person-years in 2011 and remaining fairly steady up to 2013. Similar patterns were seen when initiation rates were stratified by sex, age group, and Townsend deprivation score quintile.

It is apparent that the use of statins became more widespread into the 2000s, especially as increasing evidence for the effectiveness of statins for the primary prevention of CVD became widespread.^1^–^6^ In addition, public awareness of the importance of low-density lipoprotein cholesterol reduction and the use of statins, through the media and health promotion campaigns, may have been responsible for a general increase in the uptake of statins over time.^37^ Furthermore, the cost of prescribing statins has decreased over time, which may also have influenced in the increase in statin therapy initiation.^38^,^39^

The peak in statin therapy initiation observed in 2006 coincides with the NICE recommendation that statins should be routinely prescribed for adults with clinical evidence of CVD and adults considered to be at risk of CVD and, with this, the adoption of the general rule to prescribe statins to those whose risk of developing a CVD event within 10 years exceeds 20%. As a result, the slow decline in statin initiation rates beyond 2006 may be because a large proportion of patients who were eligible for statin therapy had received their first statin prescription at this point. Another factor that may have influenced the decline in statin therapy initiation since 2006 may be the increase in the reporting of the possible adverse effects of statins.^40^–^42^

We saw evidence of a difference in statin initiation rates for males and females in recent years, with females initiating statins at lower rates than males. The lower rates for females may originate from the fact that CVD risk scores, such as the Framingham risk score or QRISK score,^9^,^10^ are usually calculated differently for males and females and, thus, males may be more likely to attain a 10-year risk score >20% and be prescribed statins. It is well known that CVD events tend to occur later for females than for males and that there are differences in the prevalence of CVD risk factors.^43^–^46^ After accounting for age and sex, statin therapy initiation rates increased as Townsend score quintile increased, thereby implying that the initiation rate of statin therapy increases as the level of social deprivation increases. This concurs with evidence from another recent study on statin therapy initiation incidence using THIN.^47^

We observed higher statin therapy initiation rates for patients aged 70–74 years, which we would naturally expect according to the NICE prescription guidelines for statins. In addition, the low initiation rates seen for age groups under 50 would generally be expected as these are age groups whom we would expect to be at the least risk of experiencing a CVD event. Generally, the 70–74 years age group had a higher statin therapy incidence rate over time than both the 60–69 years group and the 75–84 years group. It is possible that many patients who reach 75 years may already have initiated statin therapy and those in the 75–84 years age group who remain eligible for a first statin prescription represent a unique, and perhaps more healthy, subgroup of the general population. In addition, there have been a limited number of randomized controlled trials of the efficacy of statins for low-density lipoprotein cholesterol reduction in the over 75s and it may be that GPs have not always had clear guidance on statin prescriptions for older age groups.^48^ This may also explain why the statin therapy initiation rate is relatively low for the 85 years and over age group.

Statin prescription prevalence rates have increased smoothly from 1995 to 2001, but more rapidly from 2001 to 2009. In recent years, males have had a higher prescription prevalence rate than females. Again, this is possibly because males tend to be at a higher risk of CVD development than females. Prescription prevalence rates are broadly similar over time for the different Townsend score quintiles, indicating that GP registered patients appear to be receiving statin prescriptions at similar rates across all levels of socioeconomic deprivation, though there is slightly more variation in rates for recent years than in the years up to 2007.

We saw smoothly increasing prevalence rates for the 70–74 years and 75–84 years age groups up to 2009–2010, at which point the rate for the 75–84 years group became higher than that for the 75–84 years group. This effect is perhaps because patients who were prescribed statins in their early 70s (the group with the highest statin therapy initiation rate) have aged and gradually moved into a higher age group over time.

The observed increase in statin prescription prevalence from 1995 to 2013 is perhaps what would be expected when comparing our results to those from some previous studies. Both Teeling et al^17^ and Walley et al^18^ reported significant increases in the use of statins in Ireland and across Europe, in the late 1990s and early 2000s, though we note that these studies did not focus solely on the prescription of statins for the primary prevention of CVD. In our work, we have seen that the use of statins has increased even more rapidly between 2003 and 2013.

### Strengths and limitations

First, we note that this work is the most recent and comprehensive study on statin prescription rates among patients who are yet to experience a CVD event using a large database of UK primary care records.

A clear strength of this study is the large sample size which reflects the general UK population and allows us stratify quantities of interest without the problem of small group sizes for some strata. In addition, since most GPs generate prescriptions electronically, the nature of the THIN database allows us to accurately monitor statin prescriptions in a detailed manner, by patient, longitudinally. We feel that this has allowed us to make an informed and comprehensive assessment of statin prescription rates for primary prevention of CVD over time, within the UK population.

However, there are limitations to our methods and sample. We used data from those who are registered and received statin prescriptions from a GP and hence we will not have captured those who receive treatment outside primary care. We note, though, that the majority of the UK population are registered with a GP.^49^ Some individuals may be initiated treatment while they are in hospital; however, the prescribing budget lies within primary care. Also, low dose statins can be bought over the counter and we would not have captured these in our estimates.

Finally, it is important to note that receipt of a statin prescription does not necessarily imply that a patient actually takes the medication. However, we note that the large number of patients who receive repeat prescriptions for statins over time is perhaps an indication that these patients are actually taking the treatment prescribed on a regular basis.

### Clinical implications

One clinical question of interest is whether or not statins are under- or over-prescribed. Our results would allow clinicians to assess whether or not the observed statin prescription rates in THIN data are in line with those that would be expected for a particular group within the general population who have yet to experience a CVD event. As an example, results from Abramson et al^50^ suggested that, in 2011, if CVD risk was estimated using the QRISK2 score, then one would expect 2% of females and 9% of males in their 50s and 16% of females and 48% of males in their 60s to be eligible for regular statin prescriptions. Comparatively, our 2011 estimated statin prescription prevalence rates would suggest that, approximately, statins were over-prescribed for the 50–59 age group but were approximately in line with what would be expected for the 60–69 years age group.

### Final conclusion

The uptake of statins for the primary prevention of CVD within UK primary care has increased greatly over time up to 2013 with statins being more commonly prescribed to older patients in general and, in recent years, males appear to have been prescribed statins at higher rates than females. After accounting for sex and age group, the rate of statin therapy initiation is slightly higher as social deprivation level, as measured by Townsend score quintile, increases.

## Figures and Tables

**Table 1:** Demographics of the cohort for the initiation of statin therapy study

Total number of patients		6,732,469
Sex	Male	3,211,813 (47.7%)
	Female	3,520,656 (52.3%)
Townsend score quintile	1 (least deprived)	1,566,745 (23.3%)
	2	1,384,301 (20.6%)
	3	1,444,198 (21.4%)
	4	1,359,639 (20.2%)
	5 (most deprived)	977,586 (14.5%)
Mean age at start of observation		39.2 years

**Table 2:** Table showing statin therapy initiation rates for all patients and for males and females separately from 1995 to 2013 per 1,000 person-years

Year	Statin therapy initiation rate (per 1,000 person years)		
	All patients	Males	Females
1995	0.51	0.51	0.52
1996	1.44	1.29	1.59
1997	1.97	1.87	2.06
1998	2.31	2.24	2.37
1999	3.09	2.93	3.24
2000	4.28	4.29	4.27
2001	6.39	6.60	6.19
2002	9.07	9.39	8.77
2003	11.92	12.61	11.27
2004	16.32	16.66	15.99
2005	16.55	17.39	15.76
2006	19.83	21.24	18.49
2007	16.38	17.98	14.86
2008	16.14	17.88	14.50
2009	15.25	17.43	13.21
2010	12.45	14.11	10.91
2011	10.88	12.32	9.54
2012	11.38	12.66	10.20
2013	10.76	11.87	9.74

**Table 3:** Results from univariable and multivariable analyses, estimating the relative statin therapy initiation rates, adjusted for time, with regard to sex, age group, and Townsend score quintile

Variable		Relative statin initiation rate estimates (95% confidence interval)	
		Univariable model	Multivariable model
Sex	Male	1	1
	Female	0.85 (0.846, 0.855)	0.76 (0.762, 0.772)
Age group (years)	18–34	0.03 (0.029, 0.031)	0.029 (0.028, 0.029)
	35–49	0.29 (0.286, 0.291)	0.283 (0.281, 0.286)
	50–59	1	1
	60–69	1.96 (1.949, 1.979)	1.99 (1.973, 2.002)
	70–74	2.44 (2.414, 2.462)	2.48 (2.456, 2.504)
	75–84	1.54 (1.522, 1.554)	1.57 (1.552, 1.585)
	≥85	0.66 (0.647, 0.676)	0.69 (0.675, 0.705)
Townsend score quintile	1 (least deprived)	1	1
	2	1.03 (1.019, 1.036)	1.05 (1.039, 1.056)
	3	0.98 (0.975, 0.991)	1.14 (1.131, 1.150)
	4	0.98 (0.971, 0.988)	1.26 (1.251, 1.273)
	5 (most deprived)	0.99 (0.979, 0.998)	1.39 (1.372, 1.399)

**Notes:** In the multivariable model, rates for sex are adjusted for age and Townsend score, rates for age groups are adjusted for sex and Townsend score, and rates for Townsend score are adjusted for sex and age group.

**Table 4:** Demographics of the cohort for the prevalence of statin therapy study

Total number of patients		7,027,711
Sex	Male	3,356,438 (47.8%)
	Female	3,671,273 (52.2%)
Townsend score quintile:	1 (least deprived)	1,633,640 (23.2%)
	2	1,446,584 (20.6%)
	3	1,506,846 (21.4%)
	4	1,417,595 (20.2%)
	5 (most deprived)	1,023,046 (14.6%)
Mean age at start of observation		39.8 years

**Table 5:** Table showing statin prescription prevalence rates for all patients and for males and females separately from 1995 to 2013 (per 1,000 person-years)

Year	Statin prescription prevalence rate (per 1,000 person years)		
	All patients	Males	Females
1995	2.36	2.47	2.25
1996	4.01	4.31	3.74
1997	6.27	6.75	5.82
1998	9.14	9.93	8.41
1999	12.36	13.30	11.50
2000	16.30	17.23	15.43
2001	22.41	23.69	21.21
2002	30.85	32.83	28.99
2003	41.59	44.45	38.90
2004	55.33	58.68	52.17
2005	69.94	74.26	65.85
2006	84.92	90.64	79.51
2007	96.53	103.88	89.57
2008	105.63	114.76	96.99
2009	113.57	124.33	103.37
2010	118.49	130.84	106.79
2011	121.10	134.92	108.08
2012	124.79	139.69	110.79
2013	128.03	143.95	113.12
